# Enhancing Health and Wellbeing through Immersion in Nature: A Conceptual Perspective Combining the Stoic and Buddhist Traditions

**DOI:** 10.3389/fpsyg.2017.01573

**Published:** 2017-09-12

**Authors:** Marcin Fabjański, Eric Brymer

**Affiliations:** ^1^Marcin Fabjanski's School of Philosophy, Selfoff Poznan, Poland; ^2^Institute of Sport, Physical Activity and Leisure, Leeds Beckett University Leeds, United Kingdom

**Keywords:** stoic, buddhism, well-being, nature

## Introduction

A growing body of evidence from a wide range of fields indicates that physical activity in nature improves psychological health and wellbeing (Pretty et al., 2005; Howell et al., 2013; Passmore and Howell, 2014). For example, Passmore and Howell (2014) found that both eudemonic wellbeing and hedonic tone were enhanced after a 2-week outdoor activities intervention. Mitchell (2013) found an association between the regular use of natural environments for leisure activities and a lower risk of mental health issues. Carrus et al. (2015) found that contact with green space in a school environment positively influenced cognitive performance, social behavior, and affect. Improved health and wellbeing has also been associated with feelings of emotional connection to nature (Brown and Kasser, 2005; Nisbet et al., 2011; Capaldi et al., 2015; Martyn and Brymer, 2016). For example, Martyn and Brymer (2016) found that higher nature relatedness was related to low state and trait somatic and cognitive anxiety. However, as Mitchell (2013) recognized the association between wellbeing and nature might be more complex than initially understood. Despite the number of studies showing improvements in psychological health and wellbeing through activities undertaken in the presence of nature and feelings of connection to nature we are still unclear about how the relationship between people and the natural environment enhances wellbeing (Brymer et al., 2014; Korpela et al., 2014; Brymer and Davids, 2016; Yeh et al., 2016; von Lindern, 2017). This is significant because understanding how this relationship enhances health and wellbeing is important for designing effective interventions. In this paper we present a conceptual framework for understanding *how* to enhance the wellbeing benefits of nature-based experiences by drawing on principles from Stoic and Buddhist traditions. Specifically, we consider the stoic idea of *oikeiōsis*, which Nussbaum (2009) refers to as the process of the human complex attunement to the intention of the universe and the Theravāda Buddhist concept of mind awakening as abandoning of self. Both concepts seek human wellbeing and flourishing through participation in nature. We (1) show how the philosophical understandings from the Stoic and Buddhist traditions can be combined and practically applied to understand and enhance wellbeing; and (2) describe a concise system of navigating in the world, with the aim of enhancing health and wellbeing in humans.

## Current explanations of the human-nature relationship

In recent years there has been a wealth of evidence demonstrating that engagement with the natural environment benefits human health and wellbeing (Brymer et al., 2010; Herzog and Strevey, 2015; Lymeus et al., 2017). The indication being that nature-based experiences might present a unique route to lasting and meaningful wellbeing outcomes. This has led some researchers to suggest that time spent in nature might be as effective as more traditional social, psychological, or exercise interventions (Barton et al., 2012; Lymeus et al., 2017). Currently, there are few viable theoretical explanations for the relationship between experiences in the natural environment and positive changes in psychological wellbeing (Brymer et al., 2014; von Lindern et al., 2016). Although some operational descriptions have implicated a possible role for neurophysiological mechanisms and improvements in self-control, self-mastery and exposure to positive social support and environments, no clear and testable theoretical framework has been proposed to explain how wellbeing might emerge from engaging in nature-based activities (Brymer et al., 2014; von Lindern et al., 2016).

To date, theoretical frameworks and philosophical foundations typically utilized to guide research focus either on the attributes of the person or the characteristics of the environment and include Attention Restoration Theory ART (Kaplan and Kaplan, 1989) and the Biophilia hypothesis (Wilson, 1984). ART asserts that the natural world has the capacity to reduce attentional fatigue that stems from demands placed on our cognitive resources from modern lifestyles. The natural world has the ability to effortlessly hold our attention (i.e., soft fascination) and restore our cognitive resources (von Lindern et al., 2016; Lymeus et al., 2017). The Biophilia hypothesis (Wilson, 1984) suggests that we have a primordial connection with the natural world that predisposes us to respond positively to exposure to the natural environment. However, as these frameworks do not effectively determine *how* the relationship between human beings and nature benefits human health and wellbeing they are not able to provide guidance for the health professional wishing to include nature as an intervention. Furthermore, recent research suggests that the relationship might be more complex than suggested by ART or Biophilia (von Lindern et al., 2016; von Lindern, 2017). For example, Lymeus et al. (2017) found that nature images enhanced salutogenic processes and mindfulness practice for those beginning mindfulness training. A recent meta-analysis (McMahan and Estes, 2015) suggested that the beneficial effects of the natural environment on emotional wellbeing are driven primarily by increases in positive affect, and only to a lesser extent decreases in negative affect. This finding is at odds with ART (Kaplan and Kaplan, 1989) and the focus on the reduction of negative affect as the primary mechanism for enhanced wellbeing in the context of exposure to the natural world. The authors suggest that further exploration of the relationship between the natural world and human wellbeing is needed to “help clarify the manner in which nature contributes to optimal human feeling and functioning” (McMahan and Estes, 2015, p. 6).

In addition to critical perspectives on how each of the theoretical frameworks are traditionally employed to explain research findings, critiques of the current theoretical focus on the link between nature and wellbeing point to a fundamental problem preventing the development of more nuanced understandings (Brymer et al., 2014). To date much of the research exploring the relationship between human beings and the rest of the natural world has remained in the deterministic traditions relying on the Cartesian notion of “subject” and object' (Brymer and Schweitzer, 2017). From this perceptive nature is most often considered an object for the benefit of people and understanding how benefits happen is about understanding the impact of the object (i.e., natural environment) on the subject (i.e., people) (Shanahan et al., 2016). This perspective of “nature” as the physical world outside our skin (except artificial technologically created objects) has been critiqued as fundamentally flawed (Fisher, 2002). Instead Fisher describes a notion whereby people are part of nature and therefore at a fundamental level being human is also being nature. However, the pull of modern western culture tends to focus on how we are separate from or estranged from nature as opposed to being part of nature (Fisher, 2002; Brymer et al., 2009; Brymer and Schweitzer, 2017). Natural environments are most often perceived to be “places” with minimal human interference (Vining et al., 2008). For ancient philosophers of most Greek and Eastern schools, nature includes people. Nature is understood as a process of life, of which human beings are an immanent part. Returning to nature and remembering that we are nature is essential for health and wellbeing. In the present paper we will use term “natural environment” to describe the outdoor natural environment, and “nature” to describe process of life operating both outside and inside of us.

A theoretical clarification of how nature might enhance health and wellbeing that can guide interventions for the future is urgently needed (Brymer et al., 2014; Yeh et al., 2016). In this paper we argue that often overlooked philosophical perspectives from the Buddhist and Stoic traditions provide a solid framework to guide intervention designers. To do this we first introduce the Stoic and Buddhist notions most relevant to understanding how the human–nature relationship might enhance wellbeing. Then we explicate the philosophies more deeply to demonstrate the appropriateness of the concepts. Finally, we introduce a particular way of combining the teachings from Buddhist and Stoic thought in everyday life.

## An introduction to the stoic and buddhist philosophy

Two schools of philosophical practice which perceive immersion in nature as crucial for human flourishing are the Stoic school and Buddhist school. The Stoic school was established by the Greek philosopher Zeno of Citium, in fourth century BCE and the Buddhist school was founded by Gautama Buddha, most probably in the fifth century BCE. Stoic philosophy identifies two possible existential and psychological states for human beings. These states are described as the *ordinary* person state and the *philosopher* state. From the Stoic perspective a person in the ordinary state is prone to common mistakes in thinking and condemned to suffering. Being in the ordinary state signifies a person who does not make an effort to understand the laws of nature and thus cannot achieve realization or flourishing (Nussbaum, 2009). The philosopher state of being, on the other hand, signifies a person who leads an examined life and who can flourish because of their comprehension of and subordination to the laws of nature. Understanding in this context is more than an intellectual practice. It is an embodied process of attuning to nature, which engages both mind and body, and results in what Stoics call *conversion*. To put it in words of French philosopher Pierre Hadot:

For the Stoics, it was sensible reality itself that was capable of this movement of conversion. The entire universe, living and reasonable, animated by the Logos, was endowed with a vibratory movement running from the interior to the exterior and from the exterior to the interior. Conversion of the philosophical soul was then extended to the conversion of the universe and, finally, of universal reason (Hadot, 1968, p. 981).

This means that the philosopher flourishes by attuning to the rhythms of nature, and thereby eliminating barriers between self and the natural environment.

Buddhist schools offer a similar perspective and propose that there are two radically different states possible for humans: *unenlightened* and *enlightened*. The enlightened state requires a psychosomatic effort through meditation. The ideal consequence of this meditation is a life without psychological irritations which comes about as a result of mental changes. Similar to the Stoic position this process requires attuning to nature, technically known in the Buddhist scriptures as a collection of dhammas (Gethin, 2004), where illusion and conceptual superstition are overcome. The epistemological capacity which is required to attune to the process of life is called “investigation.” Effective investigation (Pāli: *dhamma vicaya*) is attuned to the rhythms of nature. Being present in physical nature enhances such an investigation. Wooded areas are considered the most conductive environment for meditation throughout the whole of the Pāli canon. According to the Theravāda school of Buddhism, meditating on and in the natural environment facilitates a focus on “truth” as differentiated from the artificial, conventional, or conceptual. The author of the classic contemporary Buddhist phenomenology and meditation handbooks Pandita writes:

Investigation shows us the characteristics of *paramattha dhamma*, or ultimate realities, which simply means objects that can be experienced directly without the mediation of concepts (Pandita, 1992, p. 105).

For both traditions the only way to reach the state of awakening or flourishing is to surrender to natural laws. Stoicism and Buddhism propose that human flourishing is not achieved by ego expression, but rather by adjustment to the natural world, including the rhythms of the natural environment. In this paper we argue that a fusion of these similar concepts from Buddhist and Stoic philosophy provides a comprehensive picture of *how* the natural environment might enhance human wellbeing. In the following sections we expand on these concepts and show how the adjustments might be facilitated.

### Stoic thought

There are a number of fundamental Stoic principles that guide the understanding of how to achieve flourishing: *oikeiō sis* (attuning to natural rhythms of the universe), *ataraxia* (tranquility), *conversion* (return to natural balance). *Oikeiō sis* is defined by the Stoics as recognizing the dynamics of reality and drawing from it happiness and strength. Nussbaum (2009) defines *oikeiō sis* as the human adjustment to the natural rhythms of the universe, which operate at a cosmic level, a natural environment level and human consciousness level. The adjustment entails an experiential relationship with nature or going into nature in an open manner which, if successfully achieved, results in a conversion. Errors of thinking attributed to human culture in all its manifestations are considered to be the main barriers to achieving this conversion.

The notion of *oikeiō sis* refers to the original stoic notion that humanity's highest aim is to live in accordance with the nature of the universe. This also means living in accordance with virtue, understood as building essential features of character, such as courage or temperance. According to the Stoic principles human beings are particles of the Universe and a person in the philosopher state of being has a duty to act according to this truth (Diogenes, in Dorandi, 2013). *Ataraxia* describes the process of attaining tranquility by attuning to nature, even when this might feel uncomfortable. For example, while many people would like life to be stable and would like to feel safe, the Stoic argument is that this longing should be abandoned, and instead people should see reality as it is; a process of constant flux. To achieve *ataraxia* a person in the philosopher state should:

Acquire the contemplative way of seeing how all things change into one another, and constantly attend to it, and exercise thyself about this part [of philosophy]. For nothing is so much adapted to produce magnanimity (Marcus Aurelius Antoninus, cited in Long, 2007, p. 262).

Exercises as those proposed by Marcus Aurelius in the quote above are not about intellectual entertainment, but rather they are about directing our attention to nature around us and within us (Hadot, 1998, p. 129). A person in the philosopher state needs to (1) consciously direct attention to what changes in the environment rather than on what remains stable, (2) repeat this focus as often as possible in order to make it a habit, (3) repeat in thoughts or in writing, philosophical statements, which suggest that reality is changing all the time. Hadot writes:

Elsewhere, Marcus writes (V, 23): Think often of how quickly beings and events pass and disappear; for substance is like a river in perpetual flux. If, Marcus adds, we can recognize that all this flux of things and events is alien to us, then we will be “raised above the tangled web of Destiny.” To be sure, our body and our vital breath are swept along by this flux, and both our representations of things which are received into the body and our vital breath belong to this flux, because they are produced by causes outside of us (Hadot, 1998, p. 118).

The anticipated outcome is an emotional state called magnanimity, which is considered pleasant and unselfish, and involves non-attachment and acceptance of change. The same notion of directing attention to change rather than stability is often given to Buddhist adepts at particular stages of meditation, which leads to the insight technically known as an “insight into arising and passing away” (Pandita, 1992, p. 271). The insight reflects the ability to attune attention to the direct experience of the rhythms of life. In turn this leads to the subsequent “insight into dissolution” (Pandita, 1992, p. 271) or the attunement to the rhythms of life and the reality of constant flux.

### Buddhist thought

Buddhist principles have been linked to reconnecting self as part of nature (Panno et al., 2017). In Buddhism the important aspects relevant to this discussion are *bojjhaṅgā* (factors of awakening) and *dhamma* (nature). Meditative practice facilitates knowledge of the constant changing pattern of nature. The postulated psychological effect is detachment from anything (since attachment to anything results in suffering). The factors leading to the awakening are known by the common name *bojjhaṅgā* (the characteristics of awakening). These are:

1. Sati–mindfulness, clarity.
2. Dhamma vicaya–investigation (curiosity to understand unobvious connections between objects of experience).
3. Viriya–vitality.
4. Pīti–joy.
5. Passadhi–happiness,
6. Samādhi–concentration,
7. Upekkhā–equanimity.

One particular tradition within Theravāda school of Buddhism, called the Thai Forest Tradition, emphasizes the fact that all the seven factors are naturally developed through immersion in the natural environment (Fisher, 2013). Very similar psychological effects, such as relaxation, enhanced creativity and concentration, and joy are described by participants exposed to nature (Nisbet et al., 2011; Capaldi et al., 2015; Panno et al., 2017). The development of the *bojjhaṅgā*, which results in awakening (*nibbāna*), has been shown to be similar to the stoic notion of conversion. Fisher writes:

Buddha insisted humans can understand their inner workings only by dispassionately observing themselves as part of nature. If we can look at how our reactions fit into the larger context of nature we can see how desire and aversion trigger our discontent. The Buddha suggested that by understanding the natural roots of desire and aversion these forces will begin to weaken, taking discontent with them (Fisher, 2013, p. 740).

Achieving enlightenment is often referred to in Buddhist scripts as “seeing dhammas,” and “dhammas” means in the original Pāli language: “nature of a thing” or “phenomenon” (Nyanatiloka, 1997, p. 55). “Dhamma” also means Buddha's teachings. Their purpose is to facilitate attunement to the process of life, which subsequently also results in the removal of self-deception. Gethin (2004) identified that the word “dhamma” also means the truth realized by the practice of the Buddhist path. Again, as in Stoic thought, being in full attunement with nature, means full realization of human potential and the realization of absolute health and wellbeing.

## A concise system of navigating in the world based on the merge of stoic and buddhist teachings

Both Stoic and Buddhist philosophical schools turn to nature, including the natural environment, as salvation from suffering and promotion of wellbeing. They search for access to an intelligence beyond individual self and refute the importance of conceptual, conventional knowledge shared by common people involved in everyday customs and ego-based ways of thinking. Positive transformation (conversion, awakening) requires that a person transcends this common way of navigating in the world. In the following passages we; (1) pinpoint specific factors in the relationship between humans and the rest of the natural environment that enable this transformation and, (2) describe a practical process for navigating in the world which employs this knowledge in order to enhance the health and wellbeing of those who use it.

The practice that opens access to the wisdom of the Stoic and Buddhist traditions, attuning to the process of life, can be summarized as questioning *self* as our identity by studying and exercising our co-dependence with the rest of the process of life. This has been termed as getting access to “Open Source Intelligence” (OSI) (Fabjański, 2014, 2016) which describes a way of knowing that is not ego or brain focused but is a function of the whole environment. Access to this way of knowing involves all three important aspects of the ancient practices introduced above. They are:

- cognitive interventions, such as contemplations;
- exposing oneself mindfully to natural patterns and rhythms of nature;
- giving up anthropomorphic perception by developing, what we call submorphic mindfulness (definition below).

The practice of attuning to the process of life consists of all these kinds of interventions. The first and the second kind are also present in contemporary coaching and psychotherapy. Cognitive interventions such as examining our beliefs and assumptions and finding ways of thinking that enhance our wellbeing are common threads in many modern therapeutic interventions (Westbrook et al., 2008). In the practice of attunement to the process of life these interventions act as preparatory exercises, which destroy, or at least weaken the intellectual barrier between self and the process of life. This preparatory phase involves (in addition to what is offered by traditional therapies) philosophical investigations, such as considering the notion of *telos* or goal of life. The second category of intervention, exposing oneself to the natural patterns of nature, can be described as an advanced form of mindfulness. This aspect facilitates a complete awareness of what is happening in our field of experience (as in regular mindfulness) (Kabat-Zinn, 2005) and involves conscious attuning to the rhythms of nature, which can be best achieved though physical interactions with the natural environment. Unlike interventions from the first category, these interventions do not employ thinking or visualizing, but utilize exercises based on sensory perception.

The third intervention, termed submorphic mindfulness (Grec. *morphē*–form, shape), stems from meditation processes such as “The Path of Purification” and employs practices such as attending to the awareness of the four elements (fire, earth, water, and wind). This intervention requires that the participant attunes their attention to objectless, changing phenomena, such as the heat or coldness in our body (the element of fire), hardness and softness (the element of earth), moisture (the element of water), or feeling of pushing (the wind element) rather than compact objects, perceived in everyday life as separate entities, such as glass or stone or tree. Concepts and definitions are replaced by awareness of the characteristics of the elements through senses rather than verbal descriptions. A modern Buddhist meditation manual describes the process in the following way:

To discern pushing, begin by being aware, through the sense of touch, of pushing in the center of the head as you breathe in and out. When you can feel it, concentrate on it until it becomes clear to your mind. Then move your awareness to a part of the body nearby, and look for pushing there. This way you will slowly be able to discern pushing first in the head, then the neck, the trunk of the body, the arms, and the legs and feet. Do it again and again, many times, until wherever you place your awareness in the body you see pushing easily (Pa-Auk, 2000, p. 116).

Submorphic mindfulness is not just about introspection. It consists of two parts: (1) proprioceptive observing of sensations within our body, such as the four elements (earth, air, water, and fire), as well as (2) observing the same elements outside of the body in natural environments by sense perception, such as touching, hearing, and seeing. Anthropomorphic perspectives and the artificial barriers between human beings and the environment are questioned. In this way the intervention intends to facilitate the realization that the perceived division between the mental and physical, and human beings and the environment is not real. Rather these concepts are considered coupled.

In summary, what we have called the OSI hypothesis shares with the biophilia hypothesis the view that there is an instinctive bond between human beings and other living systems. But the OSI hypothesis, completely abandons the anthropomorphic perspective. While attuning to the process of life a person perceives reality, including her/his own body, as much as possible, as a part of a bigger, natural and evolutionary process. What is present in this process is human consciousness, but also bacteria, viruses, scents, and vibrations. They are all equally important. This is a process-centric view, in which the human being is seen only as a temporary sub-system of the whole. OSI transcends organismic identities of any such sub-systems, human or otherwise.

## Conclusion

A fusion of philosophical perspectives from the Stoic and Buddhist schools of thought suggests that nature might enhance health and wellbeing by facilitating a way of being that attunes to the inherent process of nature, a process similar to mindfulness. This way of being can be enhanced with specific practices designed to facilitate a deeper and more profound realization and acceptance of nature's way of being and that humans are just part of a larger ever changing process. The practice of attuning to the process of life, based on the OSI hypothesis, combines three kinds of interventions, stemming from Stoic and Buddhist schools (cognitive interventions, such as contemplations; exposing oneself mindfully to natural patterns and rhythms of nature; giving up anthropomorphic perception by developing submorphic mindfulness), which taken together facilitates a coherent system of navigating in the world.

## Author contributions

All authors listed have made a substantial, direct and intellectual contribution to the work, and approved it for publication.

### Conflict of interest statement

The authors declare that the research was conducted in the absence of any commercial or financial relationships that could be construed as a potential conflict of interest.
